# Novel organic selenium source hydroxy-selenomethionine counteracts the blood-milk barrier disruption and inflammatory response of mice under heat stress

**DOI:** 10.3389/fimmu.2022.1054128

**Published:** 2022-12-01

**Authors:** Yuhui Zheng, Yanyan Zhao, Wenjun He, Yajing Wang, Zhijun Cao, Hongjian Yang, Wei Wang, Shengli Li

**Affiliations:** State Key Laboratory of Animal Nutrition, College of Animal Science and Technology, China Agricultural University, Beijing, China

**Keywords:** hydroxy-selenomethionine, blood-milk barrier, heat stress, inflammatory response, antioxidant activity

## Abstract

Heat stress (HS) in summer has caused huge economic losses to animal husbandry production recently. When mammary gland is exposed to high temperatures, it will cause blood-milk barrier damage. Hydroxy-selenomethionine (HMSeBA) is a new selenium source with better guarantee of animals’ production performance under stress, but whether it has protective effect on heat stress-induced blood-milk damage is still unclear. We established mammary epithelial cells and mice heat stress injury models to fill this research gap, and hope to provide theoretical basis for using HMSeBA to alleviate heat stress damage mammary gland. The results showed that (1) Heat stress significantly decreases *in vitro* transepithelial electrical resistance (TEER) and cell viability (*P* < 0.01), and significantly decreases clinical score, histological score, and total alveoli area of mice mammary gland tissue (*P* < 0.01). (2) HMSeBA significantly increases TEER and fluorescein sodium leakage of HS-induced monolayer BMECs (*P* < 0.01), significantly improves the milk production and total area of alveoli (*P* < 0.01), and reduces clinical score, histological score, mRNA expression of heat stress-related proteins, and inflammatory cytokines release of heat-stressed mice (*P* < 0.01). (3) HMSeBA significantly improves tight junction structure damage, and significantly up-regulated the expression of tight junction proteins (ZO-1, claudin 1, and occludin) as well as signal molecules PI3K, AKT, and mTOR (*P* < 0.01) in heat-stressed mammary tissue. (4) HMSeBA significantly increases glutathione peroxidase (GSH-Px), total antioxidant capacity (T-AOC), and superoxide dismutase release (SOD) (*P* < 0.01) and significantly reduce malondialdehyde (MDA) expression (*P* < 0.01) in heat-stressed mammary tissue. In conclusion, this study implemented heat-stressed cell and mice model and showed that HMSeBA significantly regulate antioxidant capacity, inhibited inflammation, and regulate tight junction proteins expression in blood-milk barrier *via* PI3K/AKT/mTOR signaling pathway, so as to alleviate mammary gland damage and ensure its structure and function integrity.

## Introduction

Rising global temperatures have increased the frequency and intensity of extreme heat, thus leading to heat stress (HS) in animals, which refers to a stress response in which heat production is greater than heat dissipation. Heat stress can lead to feed intake reduction and changes in milk yield and composition of dairy cows, and it has led to great economic losses to the world’s dairy production (1, 2). Heat stress can affect the normal performance of the lactating organ of dairy cows and lead to the apoptosis and reduction of mammary epithelial cells (3), thereby reducing milk production (4).

Selenium, a vital nutritional trace element involved in composition of at least 25 selenoproteins that show catalytic or antioxidant capacities (5, 6). Common selenium sources include inorganic selenium (sodium selenate, sodium selenite, etc.) and organic selenium (selenium-enriched yeast, methionine selenium, etc.) (7–9). Hydroxy-selenomethionine (HMSeBA) is a novel selenium source that first entered the market in 2013, and it received approval as a source of selenium in beef and dairy cattle feed in the US in 2021. This form of organic selenium has higher selenium deposition effect than others; therefore, it can more effectively ensure the production performance of animals under heat stress. Previous studies have found that HMSeBA can effectively alleviate heat stress in broilers and pigs (10, 11). In addition, studies have shown that HMSeBA can be used as an efficient new selenium source for dairy cows to promote the antioxidant status of dairy cows and increase selenium content in their milk and plasma (7). For instance, Sun et al. verified that dietary supplementation of HMSeBA to cows under heat stress can effectively decreases malondialdehyde (MDA) concentration and increased total antioxidant capacity (T-AOC) capacity compared to sodium selenite (12).

The transmembrane resistance value is a commonly used parameter for assessing transepithelial permeability (13), and previous studies that have implemented cell culture models have found that heat stress increases barrier permeability and decreases transmembrane resistance values of canine and porcine kidney epithelial cells, as well as human colon adenocarcinoma cellsetc (14–16). Moreover, studies have also confirmed that heat stress cause intestinal barrier integrity injury by increasing intestinal permeability and reducing the resistance of intestinal epithelium (17, 18). This has been demonstrated in both visceral endotoxemia in rats and peripheral venous endotoxemia in primates (19, 20). Both *in vitro* and *vivo* studies in rodents have confirmed that heat stress leads to a significant increase in intestinal permeability, and in the isolated intestine, heat stress can lead to permeability increase of endotoxins or glucan (21, 22).

In mammalian mammary glands, mammary epithelial cells participate in lactation as secretory cells of the mammary gland (23). Adjacent mammary epithelial cells are interconnected to form a three-dimensional junction complex comprising of tight junctions, adherens junctions, and desmosomes (13, 24). The complex is pivotal in maintaining the impermeability of mammary epithelial cells and regulating the passage of ions and small molecules across the blood-milk barrier. Therefore, the integrity of the blood-milk barrier is a prerequisite for normal functioning of mammary gland (24). Nevertheless, heat stress effect on the blood-milk barrier and HMSeBA effect on the blood-milk barrier under heat stress are still unclear. Therefore, this study constructed *in vitro* and *in vivo* heat stress injury models with cow mammary epithelial cells and mice, respectively, to clarify the above scientific issues and explore new methods of protecting the heat stress-induced blood-milk barrier damage in dairy cows.

## Materials and methods

### Cell culture, sampling and analysis

Bovine mammary epithelial cells (BMECs) were cultivated in Dulbecco’s modified eagle medium (DMEM)-F12 medium (Thermo Fisher, Waltham, MA, USA) with 10% fetal bovine serum (FBS) (Thermo Fisher), 15 ng/mL epidermal growth factor (EGF) (PeproTech, Cranbury, NJ, USA), 1% non-essential amino acids (Thermo Fisher), 1% penicillin and streptomycin (Beyotime, Shanghai, China), and 1% insulin-transferrin-selenium (ITS, Thermo Fisher) for 2 days. Subsequently, cultivated in DMEM-F12 medium containing 5% FBS, 1% penicillin and streptomycin, and 1% ITS for 3 days. Cells were cultured in 37 °C, 5% CO_2_ and 95% relative humidity.

Monolayer BMECs heat stress model construction: BMECs were seeded in transwell (membrane area: 0.33 cm^2^, pore size: 0.4 μm; Corning, NY, USA), 96-well plates and six-well plates at 2×10^4^/well, 10^5^/well and 7.5×10^5^/well to determine the transepithelial electrical resistance (TEER), cell viability, and intercellular desmosome structure detection. When the cells were treated with heat stress, the cells were placed at 42 ± 0.5°C for 1 h and then returned to 37°C.

HMSeBA-treated heat-stressed monolayer BMECs: BMECs were seeded in transwell, 96-well plates and six-well plates at 2×10^4^/well, 10^5^/well, and 7.5×10^5^/well, respectively, for the detection of TEER, fluorescein sodium leakage, and cell viability. A total of four treatments were set as follows: Se free group (without HMSeBA and HS treatment), HS group, Se+HS group (HMSeBA treatment followed by HS treatment), Se group (HMSeBA treatment). HMSeBA (2% selenium content) was dissolved in double-distilled water to prepare a 200 μM selenium storage solution and stored at 4°C for later use. The cells were treated with selenium solution (HMSeBA 50 μM) for 2 h, and then placed at 42 ± 0.5°C for heat stress treatment as described above. HMSeBA was provided by Adisseo France SAS (Antony, France).

For TEER measurement, BMECs were seeded on transwell and measured TEER daily from the third day with a Millicell resistance system-2 (Millipore, MA, USA). In detail, cells were seeded in upper chamber of transwell (membrane: 0.33 cm^2^), 200 μL medium was added in upper chamber and 600 μL medium in lower chamber. The TEER value is subtracted from the blank (with no cells) to obtain the actual measured value and expressed as Ω•cm^2^.When it reached 400 Ω•cm^2^, four treatments were performed (25). Besides, cell counting kit‐8 kits (Beyotime) were used to detect cell viability. Fluorescein sodium leakage was detected as following method: 5 μL of fluorescein sodium was added to the upper transwell chamber for 30 min and 2 h, and then collected the supernatant from the lower chamber to detect the content of fluorescein sodium.

### Animal feeding and management

ICR pregnant mice (SPF Biotechnology Co., Ltd., Beijing, China) with same genetic background and age (56 ± 5day), similar weight (25 ± 2 g), and good health status (0.5 ± 0.5 days) were raised in an artificial climate box (25 ± 0.5°C, humidity 55% ± 5%, 12L: 12D, light time 07:00–19:00) and then housed in a single cage, and *ad libitum* access to food and water were provided. The basal diet (**Table 1**) was used to make treatment diets. An artificial climate incubator (Keelrein Instrument Co., Ltd, Shanghai, China) to generate HS (39°C) or room temperature (25°C) conditions, and the humidity was 55% ± 5%. All experimental protocols were approved by the National Institutes of Health Guide for the Care and Use of Laboratory Animals (AW61902202-1-2, China).

**Table 1:** Composition and nutrient levels of the basal diet (dry matter basis).

Items	Content
Ingredients (%)	
Corn starch	39.75
Casein-vitamin free	20.00
Maltodextrin	13.20
Sucrose oil	10.00
Soybean oil	7.00
Powdered cellulose	5.00
Multi-mineral premix^1^	3.50
Multi-vitamin premix^2^	1.00
L-cystine	0.30
Choline bitartrate	0.25
t-Butylhydroquinone	0.0014
Nutritional levels^3^, %	
Metabolizable, MJ/kg	16.26
Dry Matter	92.95
Crude protein	16.64
N-free extract	7.00
Neutral detergent fiber	3.97
Acid detergent fiber	3.54

^1^Multi-mineral premix contents: calcium (0.51%), phosphorus (0.32%), potassium (0.36%), magnesium (0.05%), sodium (0.13%), chlorine (0.22%), fluorine (1 ppm), iron (39 ppm), zinc (35 ppm), manganese (11 ppm), copper (6 ppm), iodine (0.21 ppm), chromium (1 ppm) and molybdenum (0.14 ppm).

^2^Multi-vitamin premix contents: vitamin A (4 IU/g), vitamin D3 (1IU/g), vitamin E (81.6 IU/g), vitamin K (0.29 ppm), thiamin hydrochloride (6.1 ppm), riboflavin (6.7 ppm), niacin (30 ppm), pantothenic acid (16 ppm), folic acid (2.1 ppm), pyridoxine (5.8 ppm), biotin (0.2 ppm), vitamin B12 (29 μg/kg) and choline chloride (1250 ppm).

^3^ Nutrient levels were measured values.

Heat-stress mice model establishment: Twenty-four mice were randomly allotted into 4 groups, and fed a basal diet and subjected to heat stress from 13:00 to 15:00 every day starting from the postpartum period. They were divided into four treatments according to the duration of heat stress: CON (0 day), 4 days, 8 days, and 12 days. Before the heat stress treatment, the number of offspring of each mouse was uniformly adjusted to 8 refer to the previous study (26). The offspring were removed before the heat stress every day and returned after the heat stress was over. The anus temperature and body weight were measured before and after the treatments, and their activity was scored. The mice were sacrificed by cervical dislocation on days 0, 4, 8 and 12 of the heat stress treatments, and the fourth pair of mammary glands tissue samples were collected for tissue damage scoring and hematoxylin-eosin staining for pathological section observation.

Effect of HMSeBA on mice under heat stress: Forty-eight mice were randomly distributed into 4 groups: Se free group (basal diet without HMSeBA and HS treatment), HS group, Se+HS group (HMSeBA treatment followed by HS treatment), Se group (HMSeBA treatment). Heat stress was performed from 13:00 to 15:00 every day from postpartum for 12 days. Se+HS and Se treatment (0.66 mg•kg^-1^ DM selenium using HMSeBA as the only Se source) was conducted from pregnant.

### Animal sampling and analysis

Anus temperature was measured using a laser temperature gun (Shenzhen Pacom, Kowloon City, China), and dehydration degree of mice was measured by changes in the body weight of the mice before and after the heat stress treatment based on a previous study (27). Besides, milk yield of mice during early lactation (0–4), transition (5–8), and mid-lactation (9–12) was estimated using the following formula as described previously (28). Milk production=[W_3_-W_2_+(W_1_-W_2_)/3]+[W_5_-W_4_+(W_3_-W_4_)/3]/2. In detail, pups’ body weight was recorded at 13:00 (W_1_), 16:00 (W_2_), 17:00 (W_3_), 20:00 (W_4_), and 21:00 (W_5_) at 3, 7, and 11 days of lactation. Using body weight loss of pups during separating from their mothers for 3 h to estimate metabolic energy loss. The pups were then allowed to suckle milk for one hour, and the differences in pup weight were used to estimate milk production (26).

Scores of clinical scoring and histological scoring were ranked from 1 to 5. Higher clinical score corresponded to a lower degree of activity of the mouse, and higher histological score corresponding to greater degrees of tissue damage. Additional scores of 0.5 in clinical score were added cumulatively with the presence of shivering, dyspnea, and intestinal disturbances (diarrhea or loose fecal pellets). The specific scoring standards of clinical and histological scoring refer to Johnzon et al. (29) and Liu et al. (30). In detail, for clinical scoring: 1-active, responsive, and no signs of any illness; 2-slower in reaction to stimuli, but otherwise active and healthy; 3-slow and lethargic, but still active; 4-inactive but still responsive to stimuli, albeit slowly; 5-inactive and non-responsive to any stimuli. For histological scoring: 1-absent; 2-minimal (the feature was present in scant or very small amount); 3-mild (the feature was consistently present in low numbers); 4-moderate (the feature was prominent and distinctive); 5-severe (the feature was overwhelming and normal architecture of the gland was obscured). All scoring performed by five experienced veterinary pathologists blinded to treatments.

Mammary tissue samples were collected for hematoxylin-eosin staining, immunofluorescence detection, transmission electron microscopy analysis, relative mRNA expression levels detection, inflammatory cytokines expression and antioxidant index detection. Specifically, hematoxylin-eosin staining of pathological sections of mice mammary gland samples was performed as follows: Fixed mammary tissues in 4% cell tissue fixative (Solarbio, Beijing, China) immediately after sampling, used hematoxylin-eosin to stain, and used a microscope to observe; Immunofluorescence detection of tight junction proteins (ZO-1, claudin 1, and occludin) and transmission electron microscopy analysis of tight junction structure as Zheng et al. (25); Real-time quantitative PCR was performed as following: total RNA was extracted from mammary gland tissues with Trizol reagent (TaKaRa Bio, Kusatsu, Japan), and RNA concentration and purity were determined with a NanoDrop 2000 spectrophotometer (Thermo Scientific, Waltham, MA) referred to Zhao et al. (31). The primers used in our study lists in **Table 2**, and the relative mRNA expression levels of each target gene were detected using the method of described before (32); Two milliliter of RIPA tissue lysate (Solarbio, Beijing, China) was added to each sample, centrifuged at 10000–14000 × *g* for 4 min, and removed the supernatant for inflammatory cytokines expression measurement using enzyme-linked immunosorbent assay (ELISA) with mouse ELISA kits (Thermo Fisher); The glutathione peroxidase (GSH-Px), superoxide dismutase (SOD) activity, T-AOC, and MDA content were determined using commercial detection kits (A042, A005, A001–3, and A015; Nanjing JianCheng Bioengineering Institute, Nanjing, China) according to the method provided by the manufacturer.

**Table 2:** Primer sequences for qPCR.

Gene name	Primer sequence (5’-3’)	Accession number
HSF1	F: CTGGTCCGTGTCAAGCAAGAGC	NM_001331152.1
	R: AGGATGGAGTCAATGAAGGCAGTTG	
HSP70	F: ACGCCAATGGTATCCTGAATGTGTC	NM_112093.3
	R: CAGCCTTGTACTTCTCTGCCTCTTG	
claudin-1	F: GCTGGGTTTCATCCTGGCTTCTC	NM_008689.2
	R: CCTGAGCGGTCACGATGTTGTC	
occludin	F: TTGGCTACGGAGGTGGCTATGG	NM_001360536.1
	R: CCTTTGGCTGCTCTTGGGTCTG	
ZO-1	F: AACCCGAAACTGATGCTGTGGATAG	NM_001163574.1
	R: CGCCCTTGGAATGTATGTGGAGAG	
PI3K	F: CGAAACAAAGCGGAGAACCTATTGC	NM_001020127.2
	R: TCTACCACTACGGAGCAGGCATAG	
AKT	F: TCAGGATGTGGATCAGCGAGAGTC	NM_001165894.1
	R: AGGCAGCGGATGATAAAGGTGTTG	
mTOR	F: ACCGTCCGCCTTCACAGATACC	NM_020009.2
	R: GCAGTCCGTTCCTTCTCCTTCTTG	
GADPH	F: GGCAAATTCAACGGCACAGTCAAG	NM_080369.3
	R: TCGCTCCTGGAAGATGGTGATGG	

F, forward; R, reverse. Primers were synthesized by Biotech (Shanghai) Co., Ltd.

### Statistical analysis

Using SAS 9.2 (SAS Institute Inc., NYC, USA) to analyze data and GraphPad Prism 8 (GraphPad Software, San Diego, CA, USA) to produce graphs. Two-way ANOVA in a 2×2 factorial arrangement was used to analyze the main effects of HS and HMSeBA and their interaction. Tukey’s method was used to analyze variance, *P* ≤0.01 was defined as extremely significant difference, 0.01 < *P* ≤ 0.05 was defined as significant difference, and 0.05 < *P* < 1 was defined as a trend of significant difference.

## Results

### Effects of heat stress on the blood-milk barrier

**Figure 1:**
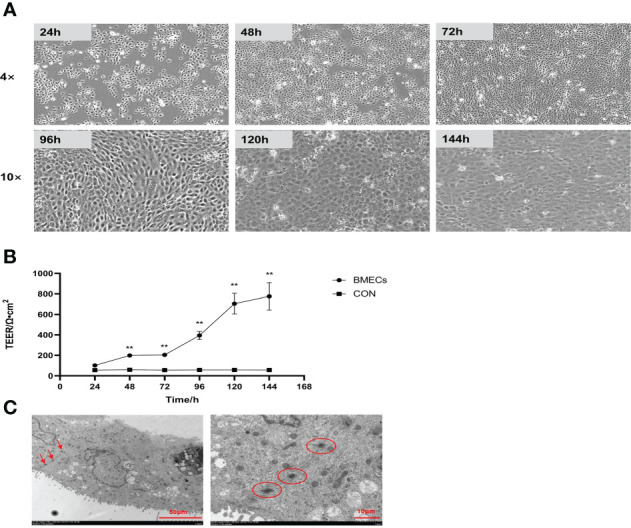
Establishment of the monolayer BMEC model (n = 9). **(A)** Morphology observations of monolayer BMECs using optical microscope; 4× and 10× indicate the magnification of the microscope objective. **(B)** Effect of culture time on the TEER of monolayer BMECs. ^**^indicates extremely significant differences between the BMECs and CON group (*P* < 0.01), and no marker indicates a non-significant difference between the BMECs and CON group (*P* > 0.05). **(C)** TEM image of the desmosome structure of monolayer BMECs. TEER means transepithelial electrical resistance. The red arrow and the circled part indicate the desmosome structure. BMECs indicates bovine mammary epithelial cells.

**Figure 2:**
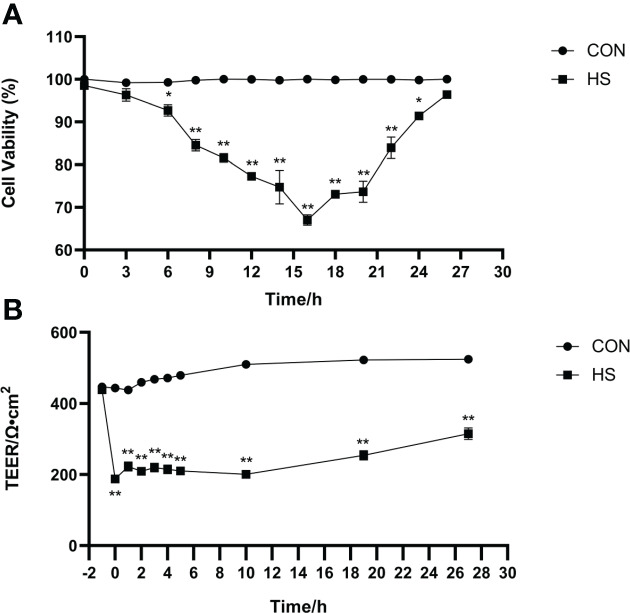
Establishment of the heat-stressed monolayer BMEC model (n = 9). **(A)** Effects of HS on cell viability of BMECs. **(B)** Effects of HS on TEER of BMECs. ^**^Indicates that the BMEC group had a very significant difference compared with CON group (*P* < 0.01). ^*^Indicates that the BMEC group had a significant difference compared with CON group (0.01 < *P* < 0.05), and no marker indicates that the BMEC group had no significant difference compared with CON group (*P* > 0.05). BMECs means bovine mammary epithelial cells. HS means heat stress. TEER means transepithelial electrical resistance.

The morphology of monolayer BMECs under a light microscope is shown in **Figure 1A**. After the cells covered the culture flask bottom, they gradually began to fuse and reached a better fusion state with blurred boundaries after 144 h. The TEER of the cells increased gradually after 24 and reached more than 700 Ω•cm^2^, which was extremely significantly higher than CON group (*P* < 0.01). (**Figure 1B**). Desmosome structure was observed between adjacent cells when the cells were cultured for 120 h under transmission electron microscopy (**Figure 1C**). The cell viability initially gradually decreased after HS, reached a minimum at 16 h, and then gradually recovered until no significant differences were observed at approximately 27 h in comparison with CON group (**Figure 2A**, *P* > 0. 05). The cell viability at 6 h and 24 h after HS was significantly lower than CON group (0.01 < *P* < 0.05), and were extremely significantly lower than CON group at other time points (*P* < 0.01). Moreover, TEER of the cells was significantly lower than the CON group (*P* < 0.01) after HS at all time points (**Figure 2B**).

**Figure 3:**
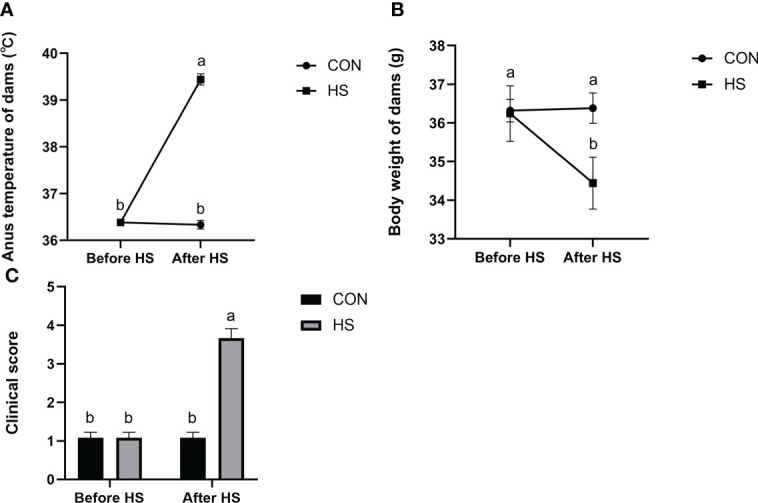
Effects of HS on anus temperature, dehydration degree, and clinical score of mice (n = 12). **(A)** Anus temperature. **(B)** Dehydration degree. Dehydration degree was described by changes in the body weight of the mice. **(C)** Clinical score. ^a-b^ Different lowercase letters indicate extremely significant differences between two groups (*P* < 0.01), while the same lowercase letters indicate no significant difference between two groups (*P* > 0.05).

The results of anus temperature, degree of dehydration (measured by body weight change) and activity score of mice before and after heat stress treatment are shown in **Figure 3**. The anus temperature was significantly higher after HS (*P* < 0.01) but did not significantly differ in CON group (**Figure 3A**, *P* > 0.05). Furthermore, the body weight and activity scores were significantly higher after HS (*P* < 0.01) but no significant difference was found before and after HS in the CON group (**Figures 3B, C**, *P* > 0.05).

**Figure 4:**
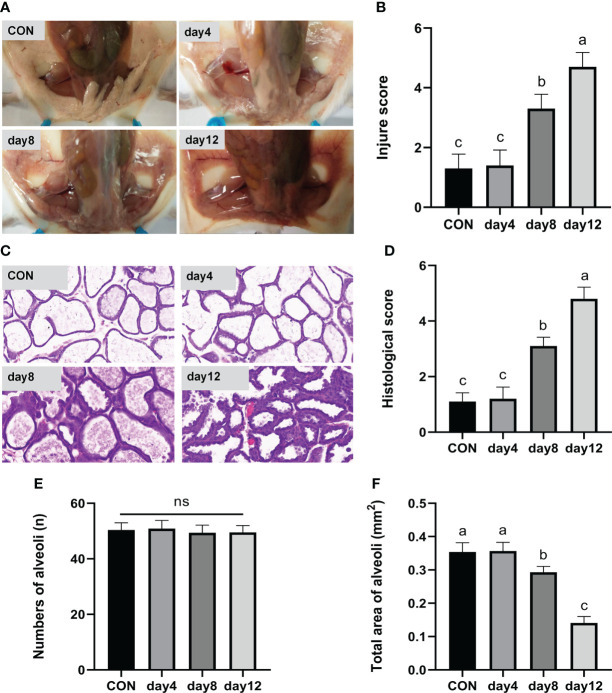
Effects of HS on mammary gland tissue of mice (n = 12). **(A)** Mammary gland tissue surface. **(B)** Clinical score. **(C)** hematoxylin-eosin-stained pathological sections. **(D)** Histological score. **(E)** Number of alveoli. **(F)** Total area of alveoli. ^a-c^ Different lowercase letters indicate extremely significant differences between two groups (*P* < 0.01), while the same lowercase letters indicate no significant difference between two groups (*P* > 0.05).

In the day 12 group (continuous heat stress treatment for 12 days, 13:00–15:00 every day) showed obvious redness and swelling, a large number of acinar walls with thickening and a severely damaged gland structure, which characterized the successful construction of a mouse heat stress mode. Specifically, the clinical conditions of the mammary gland tissue surface are shown in **Figure 4A**. The tissues presented a smooth and white surface without any redness and swelling in the CON and day 4 groups; showed slight redness, swelling and atrophy in the day 8 group; and obvious redness, swelling and atrophy was found in the day 12 group. According to the clinical score results (**Figure 4B**), an extremely significant higher score was observed in the day 12 group compared to the other groups (*P* < 0.01), and the day 8 group showed an extremely significantly higher score compared with the CON and day 4 group (*P* < 0.01), while no significant differences were observed between the CON and day 4 group (*P* > 0.05). The hematoxylin-eosin-stained pathological sections of the mammary glands are shown in **Figure 4C**. The mammary glands showed a complete acinar structure without obvious pathological changes in the CON and day 4 groups, a complete acinar structure with a small amount of acinar wall thickening in the day 8 group, and a large amount of acinar wall thickening and severe damage to the gland structure in the day 12 group. The histological scores of the mouse mammary tissue sections (**Figure 4D**) were extremely significantly higher in the day 12 group compared with the other groups (*P* < 0.01) and significantly higher in the day 8 group compared with the CON and day 4 groups (*P* < 0.01), while there was no significant difference between the CON and day 4 groups (*P* > 0.05). In addition, no significant difference in the number of acini was observed in each group (**Figure 4E**, *P* > 0.05). The total area of alveoli in the day 12 group was significantly smaller than the other groups (*P* < 0.01), the day 8 group has significantly smaller area than the CON and day 4 groups (*P* < 0.01), while no significant difference was found in the CON and day 4 groups (**Figure 4F**, *P* > 0.05).

### Effects of HMSeBA on blood-milk barrier injury induced by HS

**Figure 5:**
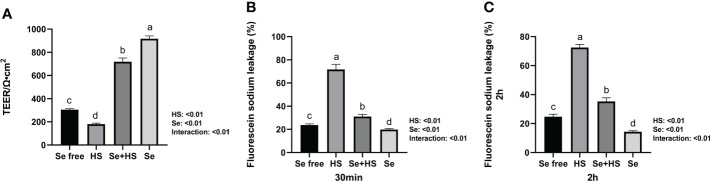
Effects of HMSeBA on permeability of monolayer BMECs under HS (n = 9). **(A)** TEER. **(B)** Fluorescein sodium leakage (30 min). **(C)** Fluorescein sodium leakage (2 h). ^a-d^ Different lowercase letters indicate extremely significant differences between two groups (*P* < 0.01), while the same lowercase letters indicate no significant difference between two groups (*P* > 0.05). BMECs means bovine mammary epithelial cells. TEER means transepithelial electrical resistance.

Se pretreatment could effectively improve the HS-induced significant increase of barrier permeability and the Se treatment could effectively decrease the barrier permeability of monolayer BMECs. In detail, both the HS and Se treatments extremely significantly affected the TEER of monolayer BMECs (*P* < 0.01), and the two had an extremely significant interaction effect (*P* < 0.01). The HS group extremely significantly reduced the TEER than the other groups (*P* < 0.01). The TEER of the Se+HS and groups was significantly higher than the HS and Se free group respectively (*P* < 0.01, **Figure 5A**). The leakage of fluorescein sodium at 30 min and 2 h are shown in **Figures 5B, C**. Both the HS and Se treatments had extremely significant effects on the fluorescein sodium permeability of monolayer BMECs at 30 min and 2 h (*P* < 0.01), and the two had an extremely significant interaction effect (*P* < 0.01). The HS group significantly increased the permeability of fluorescein sodium compared with the Se free group (*P* < 0.01); the Se+HS and Se group significantly decreased the permeability of sodium fluorescein compared with the HS and Se free groups respectively (*P* < 0.01).

**Figure 6:**
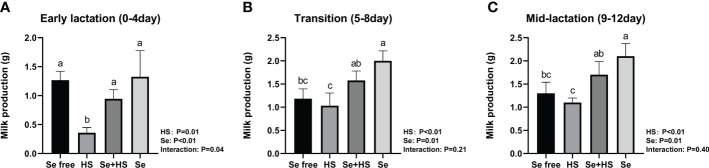
Effects of HMSeBA on milk production of mice under HS (n = 12). **(A)** Early lactation (0-4 day). **(B)** Transition (5-8 day). **(C)** Mid-lactation (9-12 day). ^a-d^ Different lowercase letters indicate extremely significant differences between two groups (*P* < 0.01), while the same lowercase letters indicate no significant difference between two groups (*P* > 0.05).

The HS and Se treatments both had extremely significant effects on the milk production of mice (**Figure 6**, *P* < 0.01); moreover, the two had a significant interaction effect on milk production during pre-lactation mice (*P* = 0.04) but have no significant interaction in transitional and mid-lactation (*P* = 0.21, *P* = 0.40). Moreover, the HS group had significantly lower milk production than the other groups during the pre-lactation period (**Figure 6A**, *P* < 0.01). In the transition (**Figure 6B**) and middle lactation period (**Figure 6C**), the milk production in the Se+HS and Se groups was significantly higher than the HS and Se frees groups respectively (*P* < 0.01).

**Figure 7:**
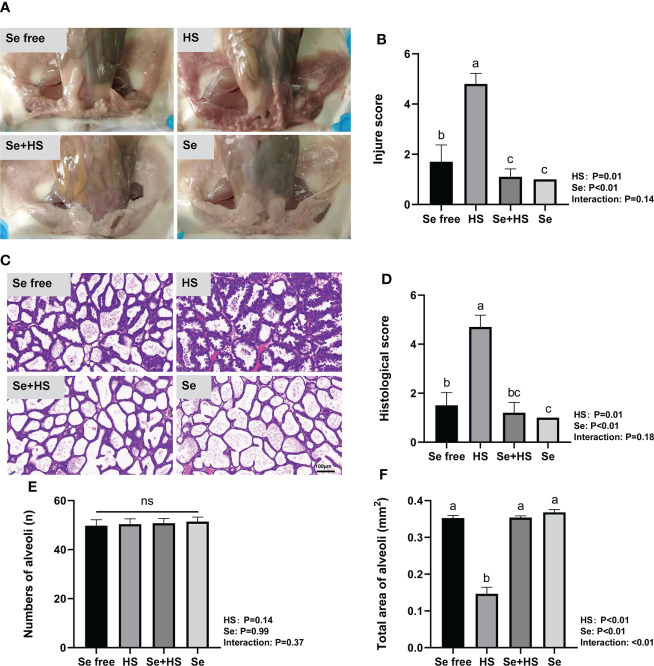
Effects of HMSeBA on mammary gland tissue of mice under HS (n = 12). **(A)** Clinical score. **(B)** Clinical score. **(C)** hematoxylin-eosin-stained pathological sections. **(D)** Histological score. **(E)** Number of alveoli. **(F)** Total area of alveoli. ^a-d^ Different lowercase letters indicate extremely significant differences between two groups (*P* < 0.01), while the same lowercase letters indicate no significant difference between two groups (*P* > 0.05). ns, no significant difference.

Dietary supplementation of HMSeBA can effectively attenuate HS-induced mammary tissue damage in mice. The mammary gland tissue surface showed slight redness and swelling in the Se free group, obvious redness and swelling in the HS group, and a smooth and white surface without any redness and swelling in the Se+HS and Se groups (**Figure 7A**). According to the clinical scores (**Figure 7B**), both the HS and Se treatments had extremely significant effects on the clinical scores (*P* < 0.01), while a significant interaction between the two was not observed (*P* = 0.14). The clinical score was extremely significantly higher in the HS group than the other groups (*P* < 0.01) and extremely significantly higher in the Se free group compared to the Se+HS group (*P* < 0.01). Whereas, no significant difference was observed in the HS and Se groups (*P* > 0.05) or Se+HS and Se groups (*P* > 0.05). The hematoxylin-eosin-stained pathological sections are shown in **Figure 7C**. The mammary gland tissue had a complete acinar structure but a small amount of acinar wall thickening in the Se free group, a large number of acinar walls with thickening and a severely damaged gland structure in the HS group, a complete acinar structure with a small amount of acinar wall thickening in the Se+HS group, the acinar structure of the mammary gland tissue of mice was intact, and there was no obvious pathological change in the Se group. According to the histological score results (**Figure 7D**), both the HS and Se treatment had extremely significant effects (*P* < 0.01), but their interaction was not significant (*P* = 0.18). The histological score of the HS groups was extremely significantly higher than Se free groups (*P* < 0.01), and Se+HS and Se groups was extremely significantly higher than HS and Se free groups respectively (*P* < 0.05). In addition, as shown in **Figures 7E, F**, there was no significant difference in the number of alveoli in the mammary gland tissue in each group (*P* > 0.05). Both the HS and Se treatments had extremely significant effects on the total area of alveoli (*P* < 0.01), and the interaction between them was extremely significant (*P* < 0.01). Total area of alveoli in the HS group was significantly smaller than the other groups (*P* < 0.01), and no significant difference was observed between the other groups (*P* > 0.05).

### Effects of HMSeBA on heat stress-related proteins and inflammatory cytokines of mice under HS

**Figure 8:**
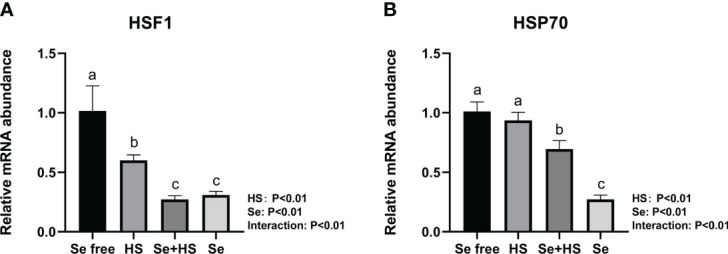
Effects of HMSeBA on the mRNA expression of heat stress related protein in mammary gland of mice under HS (n = 12). The relative mRNA expression levels of HSF1 **(A)** and HSP70 **(B)** were measured using real-time quantitative PCR. ^a-c^ Different lowercase letters indicate extremely significant differences between two groups (*P* < 0.01), while the same lowercase letters indicate no significant difference between two groups (*P* > 0.05).

Dietary supplementation of HMSeBA can effectively reduce the mRNA expression of heat stress-related proteins induced by heat stress. Under non-heat stress conditions, dietary supplementation of HMSeBA also effectively reduced the mRNA expression of heat stress-related proteins. As shown in **Figure 8**, both the HS and Se treatments had extremely significant effects on heat shock transcription factors (HSF) 1 and heat shock protein (HSP) 70 in mice mammary gland tissues, and the interaction between them was extremely significant (*P* < 0.01). The mRNA expression of HSF1 and HS70 was both extremely significantly higher in the Se free group and Se group groups than the HS and Se free groups respectively (*P* < 0.01).

**Figure 9:**
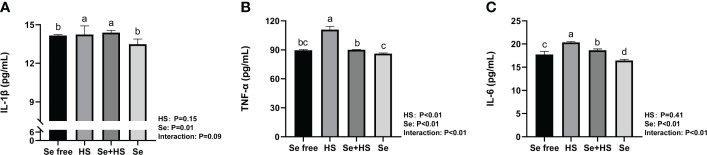
Effects of HMSeBA on the concentration of inflammatory cytokines in mammary gland of mice under HS (n = 12). The interleukin (IL)-1β **(A)**, tumor necrosis factor (TNF)-a **(B)**, and IL-6 **(C)** expression levels were measured with an enzyme-linked immunosorbent assay. ^a-d^ Different lowercase letters indicate extremely significant differences between two groups (P < 0.01), while the same lowercase letters indicate no significant difference between two groups (*P* > 0.01).

The effect of HMSeBA on the content of inflammatory cytokines is shown in **Figure 9**. The results show that the HS had extremely significant effects on the contents of TNF-α (*P* < 0.01), and Se had extremely significant effects on the contents of TNF-α and IL-6 (*P* ≤ 0.01). The interaction between HS and Se was extremely significant for TNF-α and IL-6 (*P* < 0.01), and had a significant trend on IL-1β (*P* = 0.09). The HS group had extremely significant higher content of IL-1β, TNF-α and IL-6 compared to Se free group (*P* < 0.01). The content of TNF-α and IL-6 in Se +HS group was extremely significantly lower than HS group (*P* < 0.01), while no significant difference was found for IL-1β (*P* > 0.05). The Se group had an extremely significant lower content of IL-6 than Se free group (*P* < 0.01), while no significant difference was found for IL-1β and TNF-α content (*P* > 0.05).

### Effects of HMSeBA on the tight junction proteins of mice under HS

**Figure 10:**
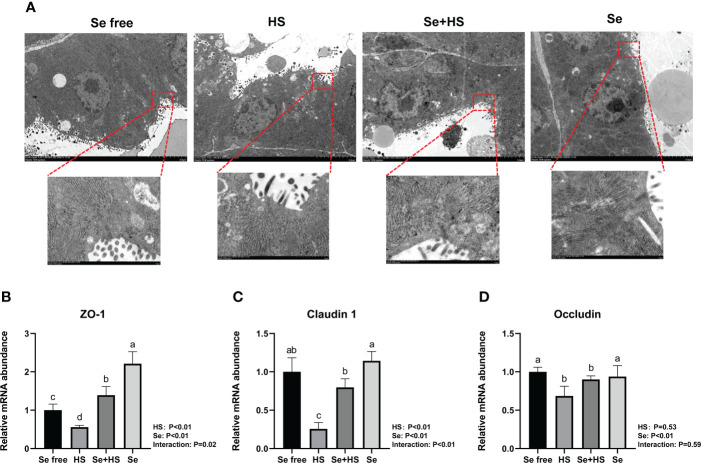
Effects of HMSeBA on the tight junction of mammary gland tissues of mice under HS (n = 12). **(A)** Tight junction structure under transmission electron microscopy. The relative mRNA expression levels of ZO-1 **(B)**, claudin 1 **(C)**, and occludin **(D)** were measured using real-time quantitative PCR. ^a-c^ Different lowercase letters indicate extremely significant differences between two groups (*P* < 0.01), while the same lowercase letters indicate no significant difference between two groups (*P* > 0.05).

The tight junction structure was observed using electron microscope (**Figure 10A**). Compared to Se free group, in the HS group, its tight junction structure was severely damaged, and was blurred under the electron microscope. The tight junction structure in the Se+HS group was obviously improved and clearly visible compared with the HS group, and was distinct clearer in the Se group than the Se free group.

The effect of HMSeBA on mRNA expression of tight junction in the mammary gland tissue is shown in **Figures 10B–D**. HS had extremely significant effect on mRNA expression of ZO-1 and claudin 1 (*P* < 0.01), while had no significant difference on occludin (*P* = 0. 53). Se had extremely significant effect on mRNA expression of three tight junction proteins (*P* < 0.01). The interaction between HS and Se was extremely significant for ZO-1 (*P* = 0.02) and claudin 1 (*P* < 0.01), and not significant for occludin (*P* = 0.59). The mRNA expression of three proteins were all extremely significant lower in HS group compared with Se free group (*P* < 0.01). Se+HS group had extremely higher mRNA expression of ZO-1 and claudin 1 compared to HS group (*P* < 0.01), while had no significant difference was found for occludin (*P* > 0.05). Se group had extremely higher mRNA expression of ZO-1 compared to Se free group (*P* < 0.01), while no significant difference was found for and claudin 1 and occludin (*P* > 0.05).

**Figure 11:**
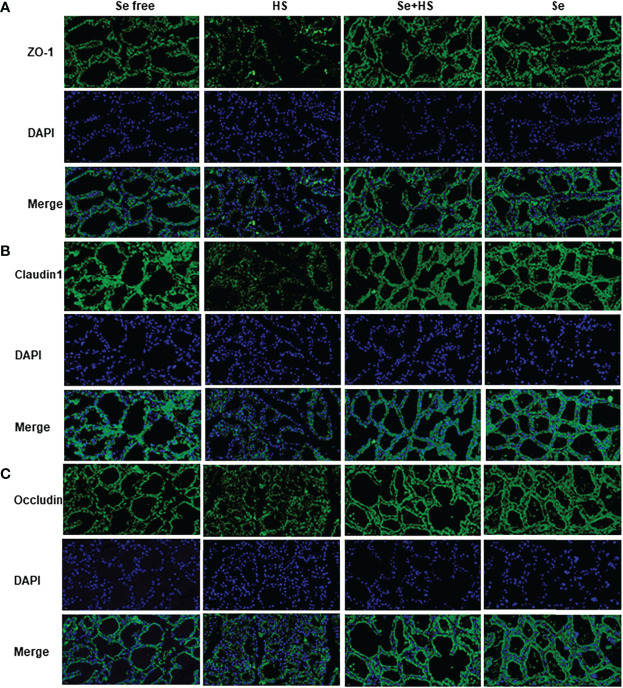
Effects of HMSeBA on the immunofluorescence of tight junction proteins in the mammary gland of mice under HS (20 ×) (n = 12). Effects of HMSeBA on immunofluorescence of tight junction protein ZO-1 **(A)**, claudin 1 **(B)**, and occludin **(C)** were determined using immunofluorescence. DAPI marks the nucleus of neutrophils, and the green label marks the tight junction proteins.

**Figure 11** shows the immunofluorescence staining of tight junction proteins in the mammary gland tissue. Dietary supplementation with HMSeBA could effectively attenuate the protein expression of three tight junction proteins induced by heat stress, and it could also effectively promote the expression of three proteins under non-heat stress conditions. The expression of three proteins in the Se+HS and Se groups was more obvious and the acinar structure was more complete and clearer in comparation with HS and Se free groups.

**Figure 12:**
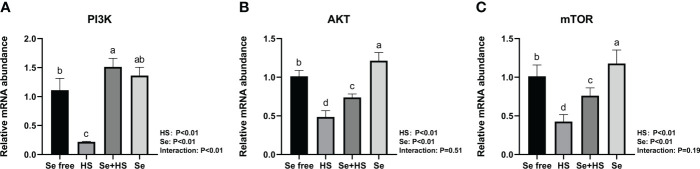
Effects of HMSeBA on the mRNA expression of PI3K/AKT/mTOR in the mammary gland of mice under HS (n = 12). The relative mRNA expression levels of PI3K **(A)**, AKT **(B)**, and, mTOR **(C)** were measured using real-time quantitative PCR. ^a-d^ Different lowercase letters indicate extremely significant differences between two groups (*P* < 0.01), while the same lowercase letters indicate no significant difference between two groups (*P* > 0.05).

The mRNA expression of the signal molecule PI3K/AKT/mTOR in the mammary glands is shown in **Figure 12**. Both HS and Se had extremely significant effect on mRNA expression of three signal molecules (*P* < 0.01). The interaction between HS and Se was extremely significant for PI3K (*P* < 0.01), and not significant for AKT (*P* = 0.51) and mTOR (*P* = 0.19). HS group had extremely lower mRNA expression of three signal molecules compared to Se free and Se+HS groups (*P* < 0.01). Se group had extremely higher mRNA expression of AKT and mTOR compared to Se free group (*P* < 0.01), while had no significant difference for PI3K (*P* > 0.05).

### Effects of HMSeBA on the antioxidant indexes of mice under HS

**Figure 13:**
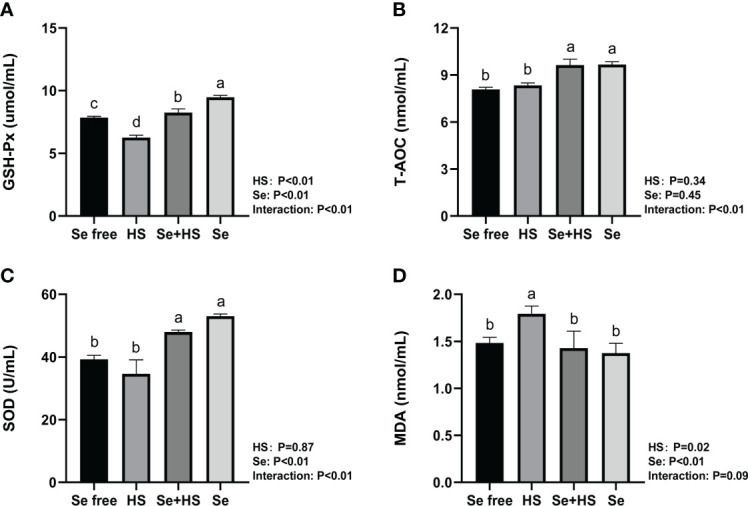
Effects of HMSeBA on the levels of antioxidant indexes in mammary gland of mice under HS (n = 12). The levels of glutathione peroxidase (GSH-Px) **(A)**, total antioxidant capacity (T-AOC) **(B)**, superoxide dismutase (SOD) **(C)**, and malondialdehyde (MDA) **(D)** were measured with an enzyme-linked immunosorbent assay. ^a-d^ Different lowercase letters indicate extremely significant differences between two groups (*P* < 0.01), while the same lowercase letters indicate no significant difference between two groups (*P* > 0.05).

The effect of HMSeBA on the content of antioxidant indicators in the mammary gland tissue is shown in **Figure 13**. HS had extremely significant effect on expression of GSH-Px (*P* < 0.01) and MDA (*P* = 0.02), while had no significant difference on T-AOC (*P* = 0.34) and SOD (*P* = 0.87). Se had extremely significant effect on expression of GSH-Px, SOD and MDA (*P* < 0.01), while had no significant difference on T-AOC (*P* = 0.45). The interaction between HS and Se was extremely significant for GSH-Px, T-AOC and SOD (*P* < 0.01), and significant trend was found for MDA (*P* = 0.09). GSH-Px expression was extremely significant higher and MDA expression was extremely significant lower in HS group than Se free group (*P* < 0.01), while T-AOC as well as SOD expression had no significant difference between two groups (*P* > 0.05). The expression of GSH-Px, T-AOC and SOD was all extremely significant higher in Se+HS group than HS group (*P* < 0.01), and the expression of MDA was significant lower in Se+HS group than HS group (*P* < 0.01). Se group had extremely significant higher expression of GSH-Px, T-AOC and SOD compared with Se free group (*P* < 0.01), while had no significant difference on MDA expression (*P* > 0.05).

## Discussion

Heat stress can lead to increased body temperature, increased respiratory rate, depression, and aggression in dairy cows, and it can also lead to poor health and decreased biological functions, such as decreased milk production and delayed estrus cycles (33). However, the specific mechanism underlying heat stress in dairy cows, especially on the mammary glands, which are the lactating organs of dairy cows, has not yet been clarified. In this study, a heat stress injury model of monolayer BMECs was successfully constructed under *in vitro* conditions (i.e., continuous culture at 37°C for 144 h, and then heat stress at 42°C for 1 h). Thus, under *in vitro* conditions, the viability of cow mammary epithelial cells decreased gradually after heat stress, reached a minimum at 16 h, and then gradually recovered until no significant differences relative to the CON group were observed at approximately 27 h. This result is consistent with Zou et al. (34), who showed that heat treatment of cow mammary epithelial cells at 42.5°C for 1 h decreased cell viability by 30% at 12 h. This may be due to the fact that heat stress causes cells to produce excess reactive oxygen species, which overload the antioxidant defense capacity of cells and induce oxidative stress. Moreover, these changes disrupt normal functions and causes cell death through apoptosis or necrosis. Previous studies have confirmed that reactive oxygen species production and oxidative stress are closely related to heat stress-induced apoptosis (35, 36), which has been demonstrated in both cow mammary epithelial cells and ovarian granulosa cells (34, 37, 38).

Transmembrane resistance is a generally accepted quantitative technique for measuring the tight junction integrity in monolayer cell culture models and represents a powerful indicator for assessing cellular barrier integrity. Some of the barrier models characterized using this metric include blood-brain barrier, gastrointestinal, lung, placenta, nose, vagina, eye, and skin models (39–46). The advantage of this method is that it is non-invasive and can be used to monitor various stages of living cells’ growth and differentiation (47). This study observed that the TEER of the cells increased gradually after 24 h of culture, increased rapidly from 72 to 120 h, and then gradually stabilized and reached more than 700 Ω•cm^2^ at 144 h of culture. This finding is consistent with previous studies. Meanwhile, the desmosome structure of monolayer cells was detected by TEM in this study, which also confirmed the successful construction of the monolayer dense bovine mammary epithelial cell model. Predecessor’s researches have shown that human and bovine mammary epithelial cells polarize when cultured on permeable transwell supports to form an epithelial barrier, that is, monolayer cells are in a state of high transmembrane resistance (TEER > 600 Ω•cm^2^) (25, 48). In addition, our study showed that the TEER of the cells was significantly lower than CON group after 1 h of heat stress but then recovered, and the difference was extremely significant compared with the CON group. This result is also consistent with the previous studies, which have demonstrated that heat stress treatment decreases TEER in models of monolayer cell barriers, such as the epithelial barrier of intestine, endothelia, blood-brain, and lung (49–52).

Our study demonstrated that heat stress caused a significant increase in anus temperature, increased dehydration, and decreased activity scores in mice, suggesting that heat stress affects mice health, which is consistent with the discover of Han et al. (26). However, heat stress effects on mice mammary gland tissue and the specific mechanisms are not yet clear. In this study, an artificial climate chamber was used to explore mouse mammary gland tissue damage with different heat stress treatment days. The results showed that continuous heat stress for 12 days (39°C and 55% ± 5% relative humidity, 2 hours per day for 12 days) can successfully build a heat stress injury model in mice, which can be used to the study the mechanism underlying the heat stress effects on mouse mammary gland tissue damage and the associated alleviation effects.

In this study, heat stress impaired the barrier permeability of monolayer BMECs and HMSeBA was able to effectively alleviate the heat stress-induced damage to the TEER and barrier permeability of monolayer BMECs. Under *in vivo* conditions, cells in the mammary glands of mice under heat stress are exposed to higher temperatures and exhibit a corresponding heat shock response. Among them, HSP production is the most accepted cellular response to heat stress (4). We found that heat stress led to mammary tissue damage and a significant reduction in acinar area and milk production in mice, while dietary supplementation of HMSeBA could significantly reduce heat stress-related proteins’ expression in mice mammary tissue and effectively attenuate heat stress damage to mammary gland tissue and milk production of mice.

Mammary epithelial cells are connected tightly by intercellular tight junction proteins that adjust paracellular permeability and play a vital role in epithelial barrier integration. Tight junctions are complex structures composed of more than fifty proteins, and we compared three tight junction-related proteins (claudin-1, occludin, and ZO-1) expression in mice mammary tissue. Among them, claudin-1 is the structural backbone of tight junctions that seals the space between two adjacent epithelial cells (53). ZO-1 could bind to other proteins and forms a scaffold or interacts with specific transmembrane proteins to anchor them into the cytoplasm (54). The first PDZ domain of ZO-1 interacts with the claudin protein (55), and decreased gene and protein expression of both proteins indicates increased permeability of the epithelial barrier. Selenium is essential in maintaining epithelial barrier function, and previous studies have demonstrated that dietary selenium supplementation could protect intestinal barrier (56), blood-brain barrier (57), and venous endothelial cell barrier (58) from stress damage by reducing oxidative stress. Our study showed that heat stress significantly reduced three tight junction proteins expression both at mRNA and protein level in mice mammary tissue, whereas dietary supplementation of HMSeBA could significantly increase claudin-1 and ZO-1 mRNA expression. This result is consistent with that of previous studies. For example, Ali et al. (59) found that dietary selenium supplementation upregulated the relative expression of claudin-1, occludin, and ZO-1 in broilers’ jejunum; Brenes et al. (60) found that selenium could enhance tight junction proteins function in human endothelial cells by up-regulating claudin, occludin and ZO-1 proteins’ expression; Tang et al. (61) proved that dietary selenium supplementation significantly reduced the expression of tight junction proteins like claudin-1 and ZO-1 at both mRNA and protein levels in the intestinal barrier under heat stress; and He et al. (62) showed that dietary HMSeBA supplementation increase occludin protein expression significantly.

The blood-milk barrier is semi-permeable, thereby allowing for only the selective transfer of components necessary for lactation, and its structural integrity prevents the uncontrolled soluble and cellular components’ exchange between blood and milk in the mammary gland. Epithelial cells are tightly connected by different structures, and tight junctions are the key to separating milk from the surrounding extracellular fluid and vascular system (24). This study showed that heat stress can damage the blood-milk barrier. The TEM, real-time PCR, and immunofluorescence results all confirmed that when mice were under heat stress, tight junction structure in the mammary gland was injured and tight junction proteins expression was significantly down-regulated. This may be related to the significant up-regulation of inflammatory cytokines expression in mammary tissue induced by heat stress. Previous studies demonstrated that cytokines also affect the blood-milk barrier and are released by leukocytes and epithelial cells during immune responses. In this study, heat stress led to a significant increase in the expression of TNF-α in mouse mammary tissue, which is consistent with Sadi et al.’s study (63), who confirmed that TNF-α can activate NF-κB and then induce increased MLCK induction, which leads to contraction of the cytoskeleton and the opening of tight junctions. The study by Sintes et al. (64) also showed that under *in vitro* conditions, mammary epithelial cells express more TNF-α mRNA if the barrier is significantly damaged. In addition, Xu et al. (65) also confirmed that IL-1β induced an increase in tight junction permeability in bovine mammary epithelial cells. The thermal environment may also affect immune system and lead to the occurrence of inflammatory responses, including changes in body temperature, behavioral and hormonal adaptation, circulatory regulation, and oxidative stress (66). This study showed that heat stress significantly increased inflammatory cytokines (IL-1β, TNF-α, and IL-6) expression in mice mammary tissue. Selenium is a micronutrient with multiple functions, such as antioxidant and immunity enhancement abilities (67, 68). Selenium deficiency can increase the risk of retained placenta, metritis, and mastitis (69, 70). Therefore, the body’s selenium status is related to maintaining homeostasis (71–73). Optimal immune function is associated with health. In this study, we found that dietary supplementation of HMSeBA significantly reduced TNF-α, IL-6, and IL-1β expression in mice mammary tissue induced by heat stress, thereby significantly reducing the heat stress-induced inflammatory factors and inducing mammary tissue damage and a significant reduction in acinar area in mice. This is consistent with previous studies, which found that selenium deficiency promotes the inflammatory responses by regulating TLR2-related pathways in mice mammary glands (74). Moreover, selenium can improve the inflammatory response of epithelial cells in dairy cows’ mammary glands by inhibiting TLR2 signaling pathways (75).

The ability of selenium to decelerate the reactions of mouse mammary tissue is closely related to its antioxidant capacity. In the antioxidant system of animals, SOD, GPx, and MDA are currently the most widely studied indicators (76). SOD eliminates superoxide radicals (O^2-^) in cells *via* a disproportionation reaction to produce H_2_O_2_ and O_2_ (77). GSH-Px is the primary antioxidant enzyme, which can reduce toxic peroxides to non-toxic hydroxyl compounds, promote H_2_O_2_ decomposition, and finally reduce free radicals’ number, and generate easily metabolized products, thereby protecting the structure and function of cell membranes from damage and interference by peroxides (78, 79). MDA is the prime product of lipid peroxidation, and the excessive accumulation of MDA will inhibit antioxidant enzymes’ activity and accelerates the oxidative damage of proteins and DNA (80). We showed that heat stress led to a significant decrease in GSH-Px expression and a significant increase in MDA expression in mouse mammary tissue, while dietary HMSeBA supplementation affect the expression of GSH-Px, T-AOC, and MDA significantly. This is consistent with the findings of Sun (7), Juniper (81), and Sun et al. (12), who also confirmed that HMSeBA has better antioxidant properties.

This study developed model of heat stress induced blood-milk barrier injury both *in vitro* and *in vivo*, and showed that HMSeBA can significantly improve the barrier permeability of HS-induced monolayer BMECs, regulate the inflammatory response, improve the body’s antioxidant capacity, and regulate the expression of tight junction proteins of the blood-milk barrier through the PI3K/AKT/mTOR signaling pathway. These changes effectively alleviated the blood-milk barrier damage induced by heat stress and ensured the structure and function integrity of the mammary gland. This study provides a novel perspective on the protective effect of HMSeBA and shows that it represents an effective prophylactic agent for maintaining blood-milk barrier function and protecting against blood-milk barrier damage under heat stress.

## Data availability statement

The original contributions presented in the study are included in the article/Supplementary Material. Further inquiries can be directed to the corresponding authors.

## Ethics statement

All experimental protocols were approved by the National Institutes of Health Guide for the Care and Use of Laboratory Animals (AW61902202-1-2, China). Written informed consent was obtained from the owners for the participation of their animals in this study.

## Author contributions

YHZ, WW, and SL conceived and designed the experiments. YHZ performed the experiments, analyzed the data and wrote the manuscript. YYZ and WH assisted to complete the sample analysis. WW and SL reviewed and edited the manuscript. WW, YW, ZC, HY and SL provided guidance for the experiments. All authors contributed to the article and approved the submitted version.

## Funding

This work was supported by National Natural Science Foundation of China (32202713) and the earmarked fund for CARS36.

## Acknowledgments

We thank the College of Animal Science and Technology Feed Engineering Technology Research Center, Yangzhou University for providing Bovine mammary epithelial cells.

## Conflict of interest

The authors declare that the research was conducted in the absence of any commercial or financial relationships that could be construed as a potential conflict of interest.

## Publisher’s note

All claims expressed in this article are solely those of the authors and do not necessarily represent those of their affiliated organizations, or those of the publisher, the editors and the reviewers. Any product that may be evaluated in this article, or claim that may be made by its manufacturer, is not guaranteed or endorsed by the publisher.
